# Bacteriological profile of neonatal sepsis and antibiotic susceptibility pattern of isolates admitted at Kanti Children’s Hospital, Kathmandu, Nepal

**DOI:** 10.1186/s13104-018-3394-6

**Published:** 2018-05-15

**Authors:** Nikita Singh Yadav, Saroj Sharma, Dhiraj Kumar Chaudhary, Prabhat Panthi, Pankaj Pokhrel, Anil Shrestha, Pappu Kumar Mandal

**Affiliations:** 1 Department of Microbiology, St. Xavier’s College, Kathmandu, Nepal; 2 Kanti Children’s Hospital, Maharajgunj, Nepal; 3 Department of Microbiology, Prithu Technical College, Institute of Agriculture and Animal Science, Tribhuvan University, Dang, Nepal; 4 Department of Microbiology, National College, Kathmandu, Nepal; 5 Department of Microbiology, Balkumari College, Chitwan, Nepal

**Keywords:** Neonatal sepsis, Bacteriological profile, Antibiotic susceptibility, Neonates

## Abstract

**Objective:**

Neonatal sepsis is a major cause of morbidity and mortality of newborns (< 1 month of age). Septicemia and drug resistance is a predominant issue for neonatal death in Nepal. This study is intended to find bacteriological profile of neonatal sepsis and antibiotic susceptibility pattern of the isolates from neonates at Kanti Children’s Hospital, Kathmandu, Nepal.

**Results:**

Out of 350 suspected cases of neonatal sepsis, 59 (16.9%) cases showed positive blood culture. The prevalent of positive blood culture with different neonatal risk factors (sex, age, birth weight, gestational age, and delivery mode) showed highest positive bacterial growth in male (52.3%); 3 or above 3 days age (71.2%); low birth weight (62.7%); preterm gestational age (31.4%); and caesarean delivery mode (63.3%). Among positive cases, the bacteriological profile was found highest for *Staphylococcus aureus* (35.6%) followed by *Klebsiella pneumoniae* (15.3%). The most sensitive and resistive antibiotics among Gram-positive isolates were gentamicin (93%) and ampicillin (78%), respectively. Meropenem and imipenem showed highest 100% effective and cefotaxime was least (28%) sensitive among Gram-negative isolates. This concludes broad ranges of bacteria are associated with neonatal sepsis and revealed variation in antibiotic susceptibility pattern among bacterial isolates.

## Introduction

Neonatal sepsis is a clinical syndrome (sepsis neonatorum) resulting from the pathophysiologic effects of local or systemic infection. It affects newborns below 1 month of age and encompasses systemic infections including meningitis, pneumonia, arthritis, osteomyelitis and urinary tract infections [1, 2]. Neonates are immune-compromised and defend weakly to bacterial infections. The bacterial agents associated with neonatal sepsis are Group B Streptococci, *Escherichia coli, Listeria monocytogenes,* coagulase-negative Staphylococci (CoNS), *Staphylococcus aureus,* Enterococci, *Klebsiella* spp.*, Enterobacter* spp., *Pseudomonas* spp., *Salmonella* spp., *H. influenzae*, *Neisseria meningitidis*, and *Streptococcus pneumoniae* [3–5].

In developing countries, unsafe birthing practices have critical role to cause neonatal infections. Globally, the neonatal morbidity and mortality cases have been estimated to 2.5–3 million, annually [6]. Neonatal mortality rate (NMR) distribution disparities can be seen based on socioeconomic, educational and geographical parameters. In Nepal, neonatal mortality has been found due to septicemia and emergence of drug resistant bacteria. According to Nepal Demographic and Health Survey (2011), 85% of total death is accounted to neonatal sepsis which is higher than previous surveys, 70% in 2006 and 69% in 2001 [7]. NMR is higher in rural areas (34 deaths per 1000 live births) than in urban areas (23 deaths per 1000 live births). Currently, emergence of multidrug resistant bacteria imposes challenges in treatment of neonatal sepsis [8, 9]. Therefore, the knowledge of prevalence of local isolates and their antimicrobial sensitivity pattern is of utmost necessary for prompt antimicrobial therapy of neonatal sepsis. This study aims to determine incidence and bacteriological profile of neonatal sepsis in relation to neonatal risk factors (sex, age, birth weight, gestational age, and mode of delivery) along with antibiotic susceptibility pattern of the isolates from neonates admitted in neonatal intensive care unit of Kanti Children’s Hospital, Kathmandu, Nepal.

## Main text

This hospital based cross-sectional study was conducted in Microbiology Laboratory of Kanti Children’s Hospital, Kathmandu, Nepal from April to September 2015. Sample size was determined based on prevalence rate of previous study [8]. A total of 350 suspected cases of neonatal sepsis were included in this study. The diagnosis of neonatal sepsis was based on clinical profile, septic screening, and blood culture.

### Methodology

The blood samples (1–2 ml) were collected from suspected neonates following standard aseptic techniques and inoculated directly into brain heart infusion (BHI; HiMedia, M210) broth containing blood in a ratio of (1:5). The culture bottles were incubated immediately at 37 °C for 5–7 days and were examined daily for growth and turbidity, hemolysis of red cells, gas bubbles and clot formation of discrete colonies. This helps in the presumptive diagnosis of positive broth culture. After incubation, subcultured from BHI broth was performed on blood agar (BA; HiMedia, M073) and MacConkey agar (MA; HiMedia, M081). The MA plates were incubated aerobically and BA plates were incubated anaerobically using BBL anaerobic jar with a GasPak™ EZ Gas Generating Container (Becton–Dickinson) at 37 °C for 24 h. The pure isolates obtained from subcultured plates were identified by following standard microbiological techniques which include studies of colony morphology, Gram-staining reactions and various biochemical properties (catalase and oxidase tests, slide and tube coagulase tests, SIM, MRVP, citrate, triple sugar iron, urease tests) [9–11].

Antibiotic susceptibility test of isolates was performed by modified Kirby-bauer disk diffusion method according to guidelines of Clinical and Laboratory Standards Institute (CLSI) [12]. The antibiotics used in this study were ampicillin (10 µg), amoxycillin (30 µg), piperacillin (100 µg), amikacin (30 µg), gentamicin (10 µg), azithromycin (30 µg), cefotaxime (30 µg), ceftazidime (30 µg), ciprofloxacin (5 µg), ofloxacin (5 µg), cotrimoxazole (25 µg), erythromycin (15 µg), meropenem (10 µg), and imipenem (10 µg). All the antibiotic discs used for susceptibility test were purchased from Himedia, India. For biochemical tests and antibiotics sensitivity tests, following reference strains were used for quality control: *E. coli* ATCC 25922; *Pseudomonas aeruginosa* ATCC 27853; *Klebsiella pneumoniae* ATCC 700603; *Salmonella typhimurium* ATCC 14028; and *Staphylococcus aureus* ATCC25923.

All the data were entered in the worksheet of SPSS software version (16.0) and *Chi* square test was performed. *P* value was calculated and considered significant only when it was less than or equal to 0.05.

### Results

**Fig. 1:**
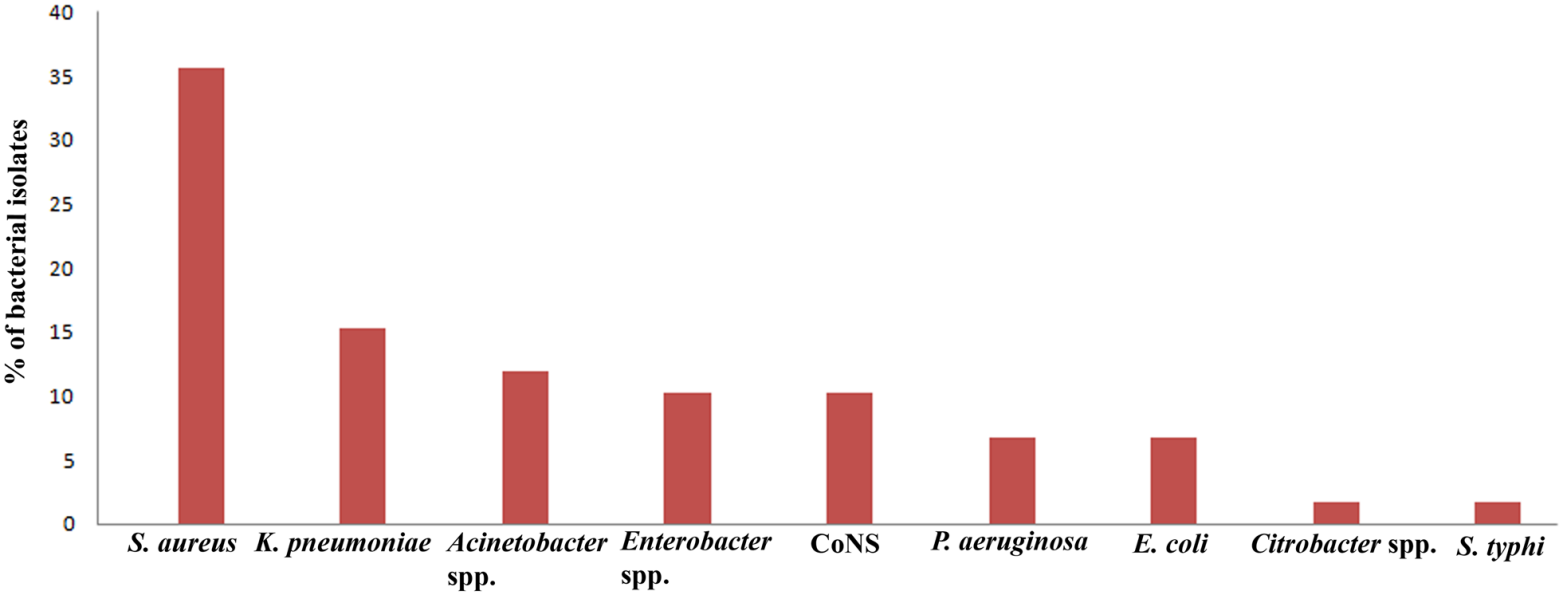
Bacteriological profile of neonatal sepsis from suspected neonates

Out of 350 blood samples, 59 samples showed growth of organism and 291 samples did not show any microbial growth. The incidence of neonatal sepsis was 16.9% among 350 blood samples enrolled in this study. Among positive cases, the bacteriological profile showed 27 (46%) were Gram-positive cocci and 32 (54%) were Gram-negative bacilli. The highest bacterial strains isolated were *S. aureus* (35.6%) followed by *K. pneumonia*e (15.3%), *Acinetobacter* spp. (11.9%), *Enterobacter* spp. (10.2%), CoNS (10.2%), *P. aeruginosa* (6.8%), *E. coli* (6.8%), *Citrobacter* spp. (1.7%), and *S. typhi* (1.7%) (Fig. 1). The highest prevalence (52.3%) of positive blood culture was found in the male neonates. There was no significant association between gender and blood culture positivity (P > 0.05). In relation to different neonatal risk factors, positive blood culture showed the highest prevalence of bacterial growth in neonatal cases with 3 or above 3 days age (71.2%); low birth weight (62.7%); preterm gestational age (31.4%); and caesarean mode of delivery (63.3%). Statistical analysis showed that there was significant association between neonatal risk and culture positivity among suspected cases (*P *< 0.05) (Table 1).

**Table 1:** Prevalence of positive blood culture in relation to different neonatal risk factors

S. no.	Neonatal risk factors	Culture positive (%)	Culture negative (%)	p value
1	Sex			> 0.05
	Male	31 (52.3)	173 (59.5)	
	Female	28 (47.4)	118 (40.5)	
2	Age			< 0.05
	Below 3 days	17 (28.8)	35 (12.0)	
	3 or above 3 days	42 (71.2)	256 (88.0)	
3	Birth weight			< 0.05
	Very low birth weight	2 (3.9)	1 (0.3)	
	Low birth wight	37 (62.7)	50 (17.2)	
	Good birth weight	20 (33.9)	240 (82.5)	
4	Gestational age			< 0.05
	Preterm	22 (31.4)	48 (68.6)	
	Term	37 (13.2)	243 (86.7)	
5	Mode of delivery			< 0.05
	Normal	21 (7.2)	269 (92.3)	
	Caesarean	38 (63.3)	22 (36.7)	

The most effective antibiotic against Gram-positive bacteria was found to be gentamicin (93%) followed by amikacin (89%) and ofloxacin (85%). The least effective drugs were erythromycin (52%) and cefotaxime (63%). Ampicillin showed the highest resistivity 78% among Gram-positive and 91% among Gram-negative isolates (Table 2). Gentamicin (90%) and ofloxacin (90%) were the most sensitive and ampicillin (76%) was the most resistive antibiotics against *S. aureus*. All the CoNS strains were sensitive towards amikacin and gentamicin. Ampicillin and ciprofloxacin showed the highest resistance (83%, each) among CoNS isolates. Among *K. pneumoniae* isolates, amikacin, gentamicin, meropenem, and imipenem were found to be 100% sensitive; and ampicillin, cefotaxime, and ceftazidime were found to be 100% resistive. Piperacillin was the most effective against all *Acinetobacter* and *P. aeruginosa* isolates. Ampicillin was 100% resistant to *Acinetobacter* strains and amoxycillin and cotrimoxazole was ineffective to all isolates of *P. aeruginosa*. Meropenem, imipenem, gentamicin, and amikacin were effective antibiotics against all the isolates of *Enterobacter*. None of the strain of *Enterobacter* showed sensitive towards amoxycillin. Ofloxacin, gentamicin, and amikacin were 100% susceptible against *E. coli*, whereas amoxycillin was resistant towards all *E. coli* isolates. Ceftazidime, amikacin, gentamicin, and ofloxacin were the most sensitive antibiotics against *Citrobacter* and *S. typhi* isolates. But, Ampicillin and cotrimoxazole showed 100% resistivity against *Citrobacter* and *S. typhi* isolates (Table 2).

**Table 2:** Antimicrobial susceptibility patterns of bacterial strains isolated from suspected cases of neonatal sepsis

Antibiotics	*K. pneumonia* n = 9 (%)	*Acinetobacter* spp. n = 7 (%)	*Enterobacter* spp. n = 6 (%)	*P. aeruginosa* n = 4 (%)	*E. coli* n = 4 (%)	*Citrobacter* spp. n = 1 (%)	*S. typhi* n = 1 (%)	*S. aureus* n = 21 (%)	CoNS n = 6 (%)
Ampicillin	0	0	NT	NT	2 (50)	0	0	5 (24)	1 (17)
Amoxycillin	5 (36)	2 (29)	0	0	0	0	1 (100)	16 (76)	3 (50)
Piperacillin	NT	7 (100)	NT	4 (100)	NT	NT	NT	NT	NT
Amikacin	9 (100)	4 (57)	6 (100)	2 (50)	4 (100)	1 (100)	1 (100)	18 (86)	6 (100)
Gentamicin	9 (100)	3 (43)	6 (100)	3 (75)	4 (100)	1 (100)	1 (100)	19 (90)	6 (100)
Azithromycin	NT	NT	NT	NT	NT	NT	1 (100)	NT	NT
Cefotaxime	0	3 (43)	1 (17)	2 (50)	2 (50)	0	1 (100)	12 (57)	5 (83)
Ceftazidime	0	1 (14)	2 (33)	2 (50)	2 (50)	1 (100)	1 (100)	NT	NT
Ciprofloxacin	5 (56)	1 (14)	1 (17)	2 (50)	3 (75)	0	1 (100)	18 (86)	1 (17)
Ofloxacin	7 (78)	4 (57)	5 (83)	3 (75)	4 (100)	1 (100)	1 (100)	19 (90)	4 (67)
Cotrimoxazole	1 (11)	5 (71)	5 (83)	0	3 (75)	0	0	NT	NT
Erythromycin	NT	NT	NT	NT	NT	NT	NT	9 (43)	5 (83)
Meropenem	9 (100)	NT	6 (100)	NT	NT	1 (100)	NT	NT	NT
Imipenem	9 (100)	NT	6 (100)	NT	NT	1 (100)	NT	NT	NT

### Discussion

Neonatal bacterial sepsis is a major cause of death in developing countries like Nepal. The emergence of antibiotics resistant bacteria and its dissemination is exacerbated by inappropriate antimicrobial consumption and precarious living condition. The most common organisms associated with neonatal sepsis vary with time of infections and geographical location [8]. Therefore, information on bacteriological profile of neonatal sepsis and effective antimicrobials for its treatment are important to combat with neonatal morbidity and mortality issues.

This study found 16.9% of neonates had microbiologically confirmed sepsis. The incidence rate of this study is quite lower compared to previous studies which have reported the incidence rate above 40% [8, 13]. The low incidence rate is due to the fact that this study was conducted in tertiary care hospital where most of the cases were referred from other hospital and clinics. Most of these referred cases had the history of antibiotics therapy prior to referral. The finding of prevalence of positive blood culture in relation to different neonatal risk factors can be useful to determine the preventive measures for neonatal sepsis (Table 1). The higher rate of growth positivity was observed in male compared to female neonates. These finding is in agreement with previous studies [14–16]. The prevalence of positive blood culture was found to be higher in 3 or above 3 days of age (late onset of sepsis) compared to below 3 days of age (early onset of sepsis). Most of the previous studies have shown similar pattern of high prevalence of neonatal sepsis in late onset of sepsis [9, 17]. Prolonged use of invasive ventilator and catheter, failure of early breast feeding, longer use of parenteral nutrition, hospitalization, surgery, cardiovascular diseases, and respiratory infections lead to late onset of sepsis among neonates [18, 19]. Positive blood culture was observed high (62.7%) in low birth weight neonates. The low birth weight is strong neonatal risk factor that leads to cause neonatal sepsis [14]. This study showed highest infection among preterm neonates compared to term neonates. The most predisposing factors of infection in neonates are premature birth and low birth weight. Preterm neonates have 3 to tenfold higher incidence of infections than full term normal birth weight infants [6]. The neonates delivered by caesarean section showed the highest positive blood culture compared to normal deliver. Studies have revealed increased risk of neonatal death delivered by caesarean compared to vaginal delivery [20].

The bacterial profile revealed the highest prevalence of *S. aureus* followed by *K. pneumoniae Acinetobacter* spp., *Enterobacter* spp., CoNS, *P. aeruginosa*, *E. coli*, *Citrobacter* spp., and *S. typhi* (Fig. 1). These bacterial strains are predominant causative agents which have been identified by several studies [8, 9, 11, 13, 15]. This study has shown the frequency of isolation of Gram-negative bacteria was higher compared to Gram-positive bacteria. The causative organism varies due to geographical area. Neonates have high chance to acquire large proportion of vaginal Gram-negative bacteria [8]. Among the Gram-positive isolates, *S. aureus* is predominant hospital acquired organism. Furthermore, *S. aureus* has greater chance of transmission from health care workers and relatives to neonates [21]. Among Gram-negative isolates, *K. pneumoniae* accounts the highest which causes infection in neonates. This finding was similar with previous studies conducted among neonates [22, 23].

The antibiotic susceptibility pattern of bacterial isolates from blood culture showed the maximum susceptibility towards amikacin, gentamicin, ciprofloxacin, and ofloxacin. However, the isolates showed the higher resistivity pattern towards ampicillin and amoxycillin. The most effective antibiotics against predominant isolates *S. aureus* and other CoNS isolates were amikacin, gentamicin, ciprofloxacin, and ofloxacin. For *K. pneumoniae* and other Gram-negative strains, meropenem, imipenem, amikacin, gentamicin, ciprofloxacin, and ofloxacin were drug of choice for treatment of neonatal sepsis. The antibiotic sensitivity test of bacterial strains isolated from this study provides insight for selection of appropriate drugs for further control of neonatal mortality rate. Ampicillin and amoxycillin which have been revealed as ineffective drugs might be due to emergence of antimicrobial genes in bacteria and inappropriate use of antibiotics prior to hospitalization of neonatal cases [8, 13].

In overall, neonatal septicemia is a life threatening emergency and its rapid treatment with antibiotics is essential. The knowledge of the etiological organisms of neonatal sepsis and their antibiotic susceptibility profile is necessary for effective therapeutic intervention. It is therefore important to note that commencement of empirical antibiotic therapy is of essence while awaiting blood culture result. The initial empiric antibiotic use must therefore be a combination of drugs to cover for the prevalent bacterial organisms in that locality.

### Conclusions

This study showed the high prevalence of *S. aureus* as Gram-positive bacteria and *K. pneumoniae* as Gram-negative bacteria among suspected neonatal cases. Overall isolates showed maximum sensitivity towards aminoglycosides and quinolones whereas minimum sensitivity towards penicillin. In Nepal, emergence of antibiotic resistance among bacterial isolates from neonatal sepsis is a major cause for treatment failure, higher morbidity and mortality. Proper antibiotic guidelines and its effective implementation could be milestone for revolution in the field of antibiotic resistance control. The epidemiology of neonatal sepsis, causative risk factors and antibiotic resistance pattern of pathogens may be used to develop guidelines for management of neonatal sepsis.

## Limitations

This study has enrolled neonates which were only admitted to Kanti Children’s Hospital, Nepal. Future research should covered suspected neonates from different part of Nepal to determine overall neonatal prevalence of the country.

## Abbreviations

BA: blood agar
CLSI: Clinical and Laboratory Standards Institute
CoNS: coagulase-negative Staphylococci
MA: MacConkey agar
MRVP: methyl-red, Voges Proskauer
SIM: sulfide, indole, motility
